# Straight Long-Range Surface Plasmon Polariton Waveguide Sensor Operating at λ_0_ = 850 nm

**DOI:** 10.3390/s20092507

**Published:** 2020-04-28

**Authors:** Yan Xu, Fei Wang, Yang Gao, Daming Zhang, Xiaoqiang Sun, Pierre Berini

**Affiliations:** 1 State Key Laboratory of Integrated Optoelectronics, College of Electronic Science & Engineering, Jilin University, Changchun 130012, China; xyan17@mails.jlu.edu.cn (Y.X.); wang_fei@jlu.edu.cn (F.W.); ygao17@mails.jlu.edu.cn (Y.G.); zhangdm@jlu.edu.cn (D.Z.); 2 School of Electrical Engineering and Computer Science, Department of Physics, and Center for Research in Photonics, University of Ottawa, Ottawa, ON K1N 6N5, Canada; pberini@uottawa.ca

**Keywords:** refractive index sensor, surface plasmon polariton, waveguide, polymer

## Abstract

A bulk refractive index sensor based on a straight long-range surface plasmon polariton (LRSPP) waveguide is theoretically designed. The waveguide sensor consists of an Au stripe that is embedded in ultraviolet sensitive polymer SU-8. The geometric parameters are optimized by finite difference eigenmode method at the optical wavelength of 850 nm. The sensitivity of 196 dB/RIU/mm can be obtained with a 1.5 μm wide, 25 nm thick Au stripe waveguide. Straight LRSPP waveguides are fabricated by a double layer lift-off process. Its optical transmission is characterized to experimentally prove the feasibility of the proposed design. This sensor has potential for the realization of a portable, low-cost refractometer.

## 1. Introduction

Surface plasmon polaritons (SPPs) are transverse magnetic polarized optical surface waves that propagate typically along the interface between a metal and dielectric (the single interface). The nanometer-thick metal stripe cladded by dielectrics with close refractive index (RI) supports the existence of long-range SPPs (LRSPPs) [1]. This bounded SPP super-mode has a symmetric field distribution, and the centimeter-long propagation distance implies a long optical interaction length for biochemical sensing [2]. However, the long propagation distance comes at the expense of weaker mode confinement, which leads to lower surface sensitivity. However, the propagation length of LRSPPs increases more rapidly than its drop in confinement, leading to a better overall sensitivity (due to a longer optical interaction length with the sensing region) compared to SPPs used in surface plasmon resonance-based detection. Beside this, LRSPPs can provide deeper penetration depth than single-interface SPPs, thus they hold promise as a probe of internal changes in biological cells immobilized on the metal surface [3]. LRSPPs can be excited by end-fire coupling with polarization-maintaining (PM) fiber or by gratings. Compact monolithic integration with microfluidic networks can be realized. Different LRSPP waveguide sensors have been reported, including Y-branch for reference sensing [4] and Mach–Zehnder interferometer with multiple outputs for perturbation-suppressed sensing [5,6], as well as multichannel waveguides for parallel biosensing [7]. In fact, a straight LRSPP waveguide can also provide analyte-receptor interaction information by simply monitoring the output power over time [8]. 

Aqueous sensing solutions with analyte typically have a low optical absorption at 850 nm [9,10,11,12,13]. Also, strong mode confinement (LRSPP) can be achieved at this wavelength and the cost of the light source and detector or camera at 850 nm is low, which is favorable to low-cost, portable sensing applications. Moreover, low-RI polymers, such as CYTOP (Asahi) or Teflon (Dupont) are commonly adopted as cladding materials for LRSPP waveguide sensors [14,15,16], because these materials have an RI that is close to water (*n*~1.33). Due to the requirement for LRSPP mode symmetry, high-RI sensing is difficult to implement with low index polymer claddings. Alternatively, the ultraviolet (UV) cured polymer SU-8 has a high RI (*n*~1.574) and has been used in the construction of SPP waveguides [17,18,19,20]. Its good physicochemical characteristics allow flexible designs and easy fabrication of LRSPP waveguide sensors, which work better for the detection of chemical reagents or mixtures detection with RIs over 1.5.

In this work, an RI sensor based on straight LRSPP waveguides operating at 850 nm is theoretically investigated. The impact of gold stripe geometric parameters on the single-mode cut-off condition, attenuation, and coupling loss are analyzed in detail. The bulk sensitivity of the proposed straight LRSPP waveguide sensor is calculated. A double layer lift-off process is adopted to fabricate LRSPP waveguides cladded by SU-8. Optical transmission measurements prove that refractometers based on LRSPP waveguides operating at 850 nm are feasible. 

## 2. Sensor Structure

**Figure 1:**
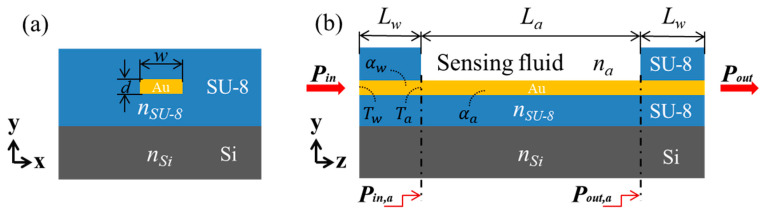
Schematic diagram of the straight long-range surface plasmon polariton (LRSPP) waveguide refractive index (RI) sensor: (**a**) Constructed by embedding the Au stripe of width *w* and thickness *d* in the polymer SU-8, and (**b**) with a microfluidic channel etched over the Au stripe to define the sensing region.

The proposed straight LRSPP waveguide RI sensor is shown in Figure 1a. A straight Au stripe of width *w* and thickness *d* is embedded in an SU-8 cladding. The Si wafer acts as the substrate to support the whole structure. The thickness of the upper and bottom cladding is 5 μm (each cladding). Figure 1b shows a longitudinal view of the proposed device. The fluidic channel of length *L_a_* is formed on the surface of the Au stripe by dissolving unexposed SU-8 in its developer. During sensing, the fluid of RI *n_a_*, which is close to that of the SU-8 cladding, flows through the channel. RI changes in the sensing solution above the Au stripe in the sensing section results in changes in the output optical power. At the operating wavelength of 850 nm, the RI of SU-8 (*n*_SU-8_) and silicon (*n*_Si_) are 1.574 and 3.643 + j0.00329, respectively [20]. The relative permittivity of Au at the wavelength of interest is −29.36 − j1.394 (which corresponds to a RI of 0.1286 + j5.420) [21]. The refractive index of the sensing solution in the fluidic channel ranges from 1.562 to 1.586, which is close to *n_SU-8_*, ensuring low-loss propagation of LRSPP mode.

## 3. Design and Optimization

### 3.1. Single-Mode LRSPP Waveguide

**Figure 2:**
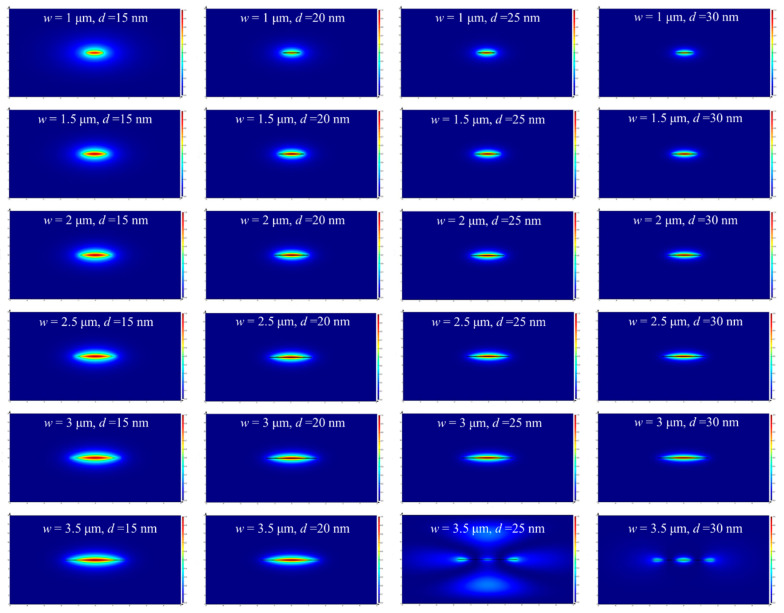
Electric field spatial distribution of Au stripe waveguide when the width *w* = 1, 1.5, 2, 2.5, 3, 3.5 μm and *d* = 15, 20, 25, 30 nm, respectively.

Appropriate mode confinement and a controllable attenuation are desirable for favorable sensing performance [22]. The geometric parameters of a straight single-mode Au stripe waveguide must be determined by computing the LRSPP mode properties numerically, and adjusting the geometry until suitable performance is achieved, at the operating wavelength of 850 nm. Here we used the finite-difference eigenmode method (FDE). The spatial distribution of the electric field of the LRSPP mode is shown in Figure 2 for the case where the sensing solution (upper cladding) was index-matched to SU-8 (lower cladding); i.e., the RIs of SU-8 and *n*_a_ were both 1.574, which resulted in symmetric RI distribution as a fully cladded case. Better mode confinement was observed as the width and thickness of the Au stripe increased; however, the propagation loss also increased with these dimensions. For different Au stripe thicknesses of *d* = 15, 20, 25, and 30 nm, the fundamental LRSPP mode is the only long-range mode supported when the Au stripe width *w* is smaller than 3 μm. As shown in Figure 2, for the case of *w* = 3.5 μm, *d* = 15 and 20 nm, the fundamental LRSPP mode is still the only long-range mode supported. When *d* = 25 and 30 nm, the high-order modes with mode power attenuations (MPAs) of 11.89 and 20.23 dB/mm, respectively, will be supported by the Au stripe. Though high-order mode sensing can be applied in dielectric-based photonic RI sensors [23,24,25], for this metal-based plasmonic RI sensing application, the analyte should be tested at the same mode condition, to avoid the interference of mode crosstalk. Therefore, the Au stripe width was chosen to be smaller than 3 μm in the following design.

### 3.2. Attenuation

As mentioned above, FDE was adopted to compute the modal properties of the LRSPP. Assuming that the LRSPPs propagate along the +*z* direction, the propagation constant *γ* can be written as *α* + j*β*. Here *α* and *β* represented the attenuation and phase constant, respectively. The LRSPPs propagated along the input and output access waveguides (*L_w_*) (both were embedded in SU-8), and the sensing waveguide section (*L_a_*) in the +*z* direction, as shown in Figure 1. The optical power output emerging from the full structure (embedded and fluidic sections) can be expressed as

(1) $$P_{out}=P_{in}T_{w}e^{-4\alpha_{w}L_{w}}T_{a}^{2}e^{-2\alpha_{a}L_{a}},$$

where *P_in_* and *P_out_* are the powers incident on the waveguide input facet and output from the waveguide, respectively, *α_w_* and *α_a_* are the mode field attenuation constants of the fully cladded and fluidic waveguides, respectively, *T_w_* is the transmittance from the optical fiber mode to the LRSPP at the input facet, and *T_a_* is the transmittance between the LRSPP mode in the sensing channel and that in the fully cladded waveguide. The mode power attenuation (MPA) in dB/mm of the LRSPP waveguide in access and sensing sections can be computed by

(2) $${\mathrm{MPA}}_{w}=20\alpha_{w}\log_{10}e$$

(3) $${\mathrm{MPA}}_{a}=20\alpha_{a}\log_{10}e$$

**Figure 3:**
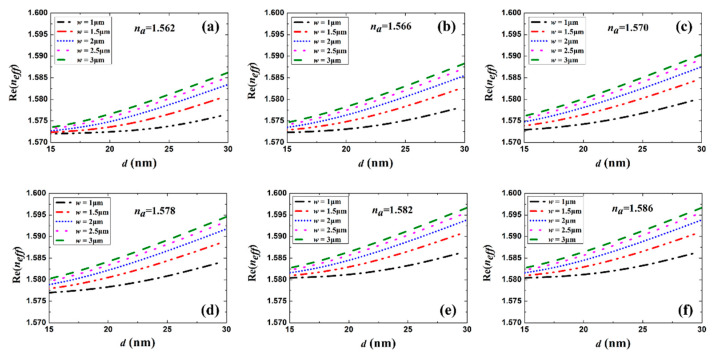
Re(*n_eff_*) as a function of Au stripe thickness *d* for different stripe widths *w* and sensing solutions with *n*_a_ of (**a**) 1.562, (**b**) 1.566, (**c**) 1.570, (**d**) 1.578, (**e**) 1.582 and (**f**) 1.586.

**Figure 4:**
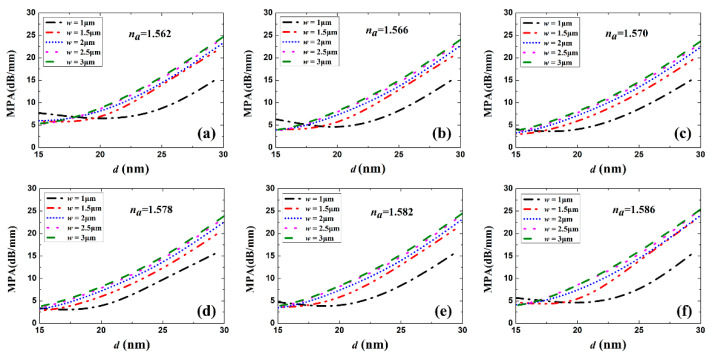
MPA as a function of Au stripe thickness *d* for different stripe widths *w* and sensing solutions with *n*_a_ of (**a**) 1.562, (**b**) 1.566, (**c**) 1.570, (**d**) 1.578, (**e**) 1.582 and (**f**) 1.586.

Figure 3 and Figure 4 show the real part of *n_eff_* (Re(*n_eff_*)) and the MPA vs. Au stripe thickness *d* for different stripe widths and sensing solution RI, respectively. Generally, Re(*n_eff_*) and MPA increased with Au stripe thickness and width, which is in accordance with LRSPP theory that thicker and wider metal stripes imply tighter mode confinement and higher loss [3]. When *w* = 1 μm, a dip appears in the MPA vs. *d* curves, which differs in character from the continuous increase in the MPA for the cases *w* = 1.5 and 2 μm. This is explained by the weaker LRSPP confinement for the case *w* = 1 μm and *d* = 15 nm. The asymmetric mode field extends more into the high-index Si substrate in this case, resulting in a higher MPA. With the increase in Au stripe thickness, the increasing mode field confinement reduced the loss caused by the Si substrate. However, thicker stripes had a higher propagation loss. Therefore, there exists a balanced thickness and a minimum MPA.

### 3.3. Coupling Loss

**Figure 5:**
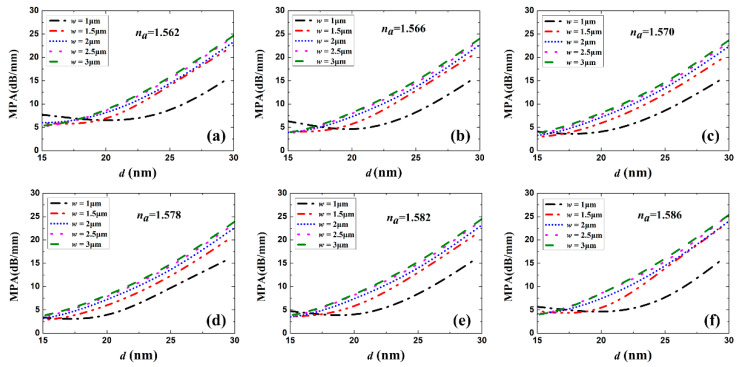
Coupling loss between cladded waveguide and sensing section as a function of Au stripe thickness *d* for different stripe widths *w* and sensing solutions with *n*_a_ of (**a**) 1.562, (**b**) 1.566, (**c**) 1.570, (**d**) 1.578, (**e**) 1.582 and (**f**) 1.586.

The coupling loss in this sensor includes the loss at the interface between the input fiber and the access LRSPP waveguide (*CL_w_*), as well as the loss at the interface between the cladded and fluidic waveguide (*CL_w_*). These losses can be expressed as:

(4) $${CL}_{w}=-10\;\times \;\log_{10}\left|T_{w}\right|$$

(5) $${CL}_{a}=-10\;\times \;\log_{10}|T_{a}|$$

where the transmittances *T_w_* and *T_a_* are given by the squared magnitude of the mode field overlap factors computed using the FDE method, following [26] (neglecting Fresnel loss due to material discontinuity). Then, the coupling loss *CL_a_* of one facet can be computed vs. Au stripe thickness *d* for different widths *w* and different sensing fluid RI, as shown in Figure 5. When the RI of the sensing fluid takes on the values of *n_a_* = 1.562, 1.566, 1.570, 1.578, 1.582, and 1.586, the symmetry of the structure is perturbed, increasing the coupling loss. For all cases of *w* and *n_a_* considered, *CL_a_* decreases with increasing *d*, up to the maximum considered (*d* = 30 nm), as the confinement of the LRSPP increases. There is no coupling loss (*CL_a_* = 0) for *n_a_* = 1.574 because the RI of the sensing solution is matched to the SU-8 cladding. When the RI of sensing fluid is lower or higher than that of SU-8, the coupling loss increases due to the distortion of waveguide RI symmetry. When *w* is larger than 1.5 μm, *CL_a_* changes little when the Au stripe thickness is over 20 nm, which is meaningful for waveguide geometry optimization. As shown in Figure 4 and Figure 5, when *d* is larger than 20 nm, a low *CL_a_* and MPA are expected for *w* = 1.5 μm. Therefore, the waveguide *w* is set to 1.5 μm in what follows.

### 3.4. Sensing Length

As has been reported, the sensing length *L_a_* is key to sensor performance [27]. The optimized sensing length can be obtained from the insertion loss (IL) in dB, expressed using Equations (1)–(5) as:

(6) $$\mathrm{IL}={CL}_{w}+2{CL}_{a}+{\mathrm{MPA}}_{w}L_{w}+{\mathrm{MPA}}_{a}L_{a}.$$

**Figure 6:**
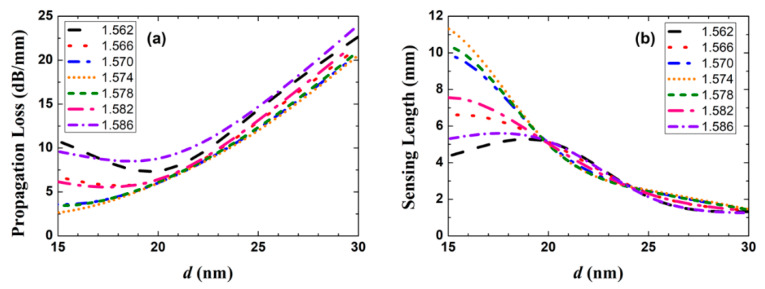
(**a**) Propagation loss (MPA*_a_*) and (**b**) sensing length (*L_a_*) as a function of Au film thickness *d* for different *n_a_*, with the width of Au stripe set to 1.5 μm.

Theoretically, a longer sensing length gives a larger output power change for the same analyte. However, the impact of background noise and performance of optical power meter determine an upper limit for the insertion loss of the fluidic section, say, MPA*_a_L_a_* = 30 dB [28]. We then plot in Figure 6 the propagation loss (MPA*_a_*) and the corresponding sensing length (*L_a_*) as a function of Au thickness *d* for different RI (setting *w* = 1.5 μm, as mentioned above). As shown in Figure 6a, when *n_a_* is close to *n_SU-8_*, MPA*_a_* increases linearly with *d*. The existence of a minimum MPA*_a_* around *d* = 20 nm for *n_a_* = 1.562, 1.566, 1.582, and 1.586 can be explained by the mode field distortion and loss induced by the high-index Si substrate. When *d* is larger than 20 nm, the propagation loss increases with the Au film thickness for different *n_a_*, which is in accordance with the theoretical prediction in Section 3.2. As shown in Figure 6b, except for *n_a_* = 1.562 and 1.586, *L_a_* decreases with increasing *d* for *d* > 20 nm. Considering Figure 4, Figure 5 and Figure 6, the propagation loss trades off against the coupling loss and the sensing length—in order to have a compact sensor, *d* is selected to be 25 nm as a compromise.

## 4. Results and Discussion

**Figure 7:**
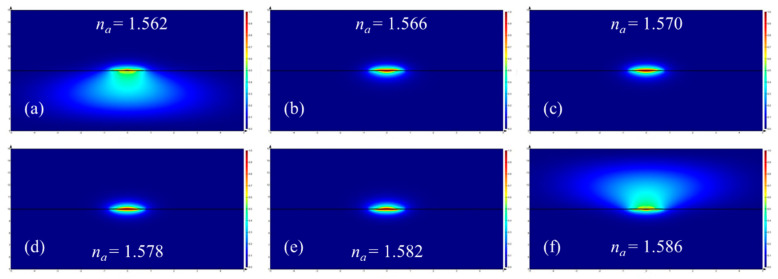
Distribution of the vertical transverse electric field component (*E_y_*) of the fundamental LRSPP mode for different cases of RI (**a**) 1.562, (**b**) 1.566, (**c**) 1.570, (**d**) 1.578, (**e**) 1.582 and (**f**) 1.586 in the fluidic channel (*n_a_*). Here, the width and thickness of the Au stripe are 1.5 μm and 25 nm, respectively.

For bulk sensing, the output power of a straight fluidic waveguide with its channel filled with a standard RI fluid can be measured. The sensitivity can be obtained from the plot of *P_out_* vs. standard RI and the slope obtained by linear fitting to the measured data. When the width and thickness of the Au stripe are 1.5 μm and 25 nm, respectively, the distribution of the vertical transverse electric field component (*E_y_*) of the fundamental LRSPP is plotted in Figure 7 for different RI of the sensing fluid. When a fluid satisfying *n_a_* < *n_SU-8_* = 1.574 fills in the fluidic channel, the mode field distribution becomes slightly asymmetric, spreading into the SU-8 cladding, because of the mismatch of RI. For a sensing fluid satisfying *n_a_* = *n_SU-8_* filling the fluidic channel, the structure becomes symmetric, and the mode is identical to that in a fully cladded waveguide, exhibiting a mode field distribution that is symmetric. For the case of a sensing fluid satisfying *n_a_* > *n_SU-8_*, the mode profile becomes increasingly asymmetric, spreading into the fluidic channel. Both asymmetric cases support LRSPPs that have a higher MPA than in the symmetric case.

**Figure 8:**
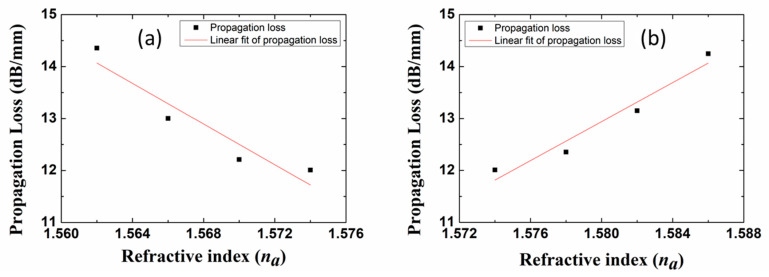
Propagation loss (MPA*_a_*) as a function of *n_a_*; (**a**) *n_a_* < *n_SU-8_*, and (**b**) *n_a_* > *n_SU-8_*.

The propagation loss (MPA*_a_*) as a function of the RI of the fluid in the sensing channel is calculated and plotted in Figure 8. We observe that the sensor exhibits different sensitivities whether *n_a_* is smaller (Figure 8a) or larger (Figure 8b) than *n_SU-8_*. In Figure 8a, the propagation loss increases with decreasing fluid RI, due to increasing mode field asymmetry (fields extending into the lower SU-8 cladding). In Figure 8b, the propagation loss increases with increasing fluid RI, also due to increasing mode field asymmetry (fields extending into the fluidic channel). According to the definition of bulk sensitivity *S*

(7) $$S=\frac{{\Delta \mathrm{MPA}}_{a}}{{\Delta n}_{a}},$$

sensitivities of 196 dB/RIU/mm and 188 dB/RIU/mm are deduced from Figure 8a,b, respectively. As shown in Figure 7, the slight sensitivity difference is induced by asymmetric mode field distribution and the Si substrate absorption when *n_a_* < *n_SU-8_*.

To better illustrate the relationship between the mode field distribution and the sensing performance, the fundamental mode power distributions in SU-8, Au-stripe, and the analyte are calculated at *w* = 1.5 μm, *d* = 25 nm. As shown in Table 1, less than 0.05% fundamental mode power distributes in the Au core, because LRSPP mode is a surface wave, and hardly penetrates into the metal. Most power distributes in surrounding dielectrics, which is in favor of the analyte detection. Since the mode field extends deeper into the dielectric, that has a higher RI, mode power in SU-8 is larger than that in the analyte, when *n_a_* < *n_SU-8_*. In addition, the asymmetry of power distribution, as well as the propagation loss, deteriorates with the progress of RI asymmetry between the upper and lower claddings. This rule also works at other Au stripe dimensions. According to LRSPP waveguide theory, better mode confinement implies less mode field spreading into the dielectric, accompanied by higher propagation loss. For sensing application, the larger the power ratio in the Au core, the higher the sensitivity that can be obtained under the same RI step variation. However, MPA increases with the improvement of sensitivity. Therefore, the compromising Au stripe dimensions of *w* = 1.5 μm and *d* = 25 nm are selected to offer the best sensitivity of 196 dB/RIU/mm.

To better evaluate the performance of the proposed sensor, we quantitatively compare the performance of the proposed sensor to those of traditional optical fiber sensors and SPP-based RI sensors. Due to the intrinsic characteristic diversity, the RI range and sensitivity of these RI sensors are inconsistent with each other. Therefore, it is hard to evaluate the performance of sensors under the same condition. Nevertheless, we try to make the comparison in terms of bulk RI sensitivity, figures of merit, and working wavelengths. As shown in Table 2 below, the proposed straight LRSPP waveguide sensor shows the highest RI detecting range, as well as the simplest structure. To be noted, compared with the experimental results in References [29,30,31,32,33,34], the data in this work is theoretically proved.

## 5. Experiment

### 5.1. Sensor Fabrication

**Figure 9:**
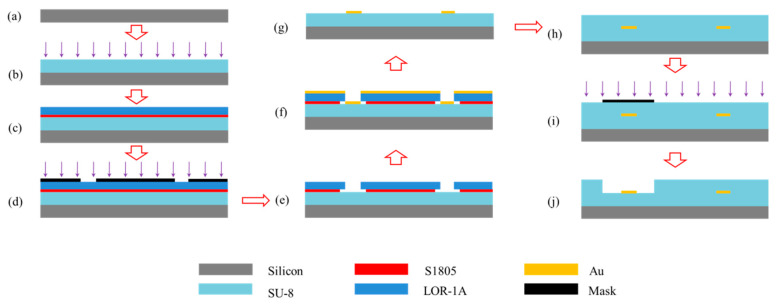
Fabrication process flow of LRSPP waveguide sensors. (**a**) silicon substrate preparation, (**b**) spin-coating and curing of SU-8 bottom cladding, (**c**) LOR-1A and S1805 stack formation, (**d**) UV photolithography, (**e**) LOR-1A and S1805 development, (**f**) Au thermal deposition, (**g**) double-layer lift-off, (**h**) spin-coating and curing of SU-8 upper cladding, (**i**) UV photolithography, (**j**) development.

A double-layer lift-off process was adopted to fabricate the LRSPP waveguide sensor [35,36]. As shown in Figure 9, SU-8 2005 (Kayaku Advanced Materials Inc., Westborough, MA, USA) was first spin-coated on the silicon substrate and UV-cured at an exposure dose of 100 mW (λ = 365 nm). Then, the lift-off resist LOR-1A (Kayaku Advanced Materials Inc., Westborough, MA, USA) and photoresist S1805 (Shipley Corp., Coventry, UK) were spun onto the SU-8 bottom cladding and thermally cured, sequentially. The time and temperature of baking were well controlled to guarantee precise development. UV exposure was conducted at a dose of 60 mJ to transfer waveguide patterns into the resist S1805. After development in MF-321 (Shipley Corp., Coventry, UK), an undercut was successfully achieved. The Au film was deposited by a thermal evaporator. A quartz crystal microbalance was used to maintain the deposition rate at 0.5 Å/s to form a smooth continuous Au film with a thickness of 25 nm. Lift-off was done with PG Remover (Kayaku Advanced Materials Inc., Westborough, MA, USA) at room temperature, leaving the defined Au features on the SU-8 bottom cladding surface.

**Figure 10:**
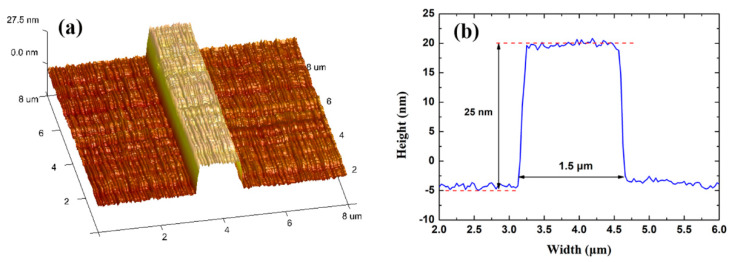
(**a**) areal scan, and (**b**) linear scan of an Au stripe on the SU-8 bottom cladding by atomic force microscopy. The Au stripe is 1.5 μm wide and 25 nm high.

**Figure 11:**
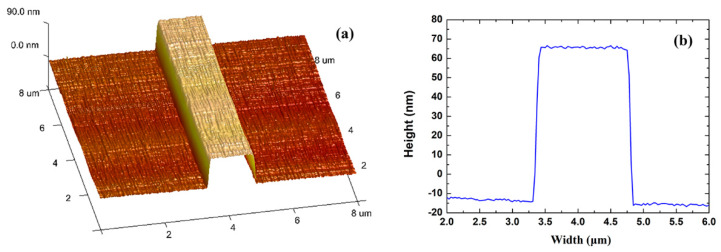
(**a**) areal scan, and (**b**) linear scan of an Au stripe by atomic force microscopy after oxygen plasma cleaning, but before application, of the SU-8 upper cladding.

The Au stripe was characterized by atomic force microscopy (AFM) after step (g). As shown in Figure 10, the Au stripe has lift-off residue on the Au surface, which leads to a root mean square roughness (*R_q_*) of 9.073 nm. To remove the residue, oxygen plasma cleaning was conducted, leading to a rectangular Au stripe with smoother surface (*R_q_* = 1.480 nm), as shown in Figure 11. The increased stripe height compared to Figure 10a originates from the oxygen plasma etching into the SU-8 bottom cladding. After step (g), the top SU-8 cladding was spin-coated and cured using the same processing parameters as in the case of the SU-8 bottom cladding. The fluidic channel was defined lithographically through development of the SU-8, exposing the Au stripe. 

### 5.2. Characterization

**Figure 12:**
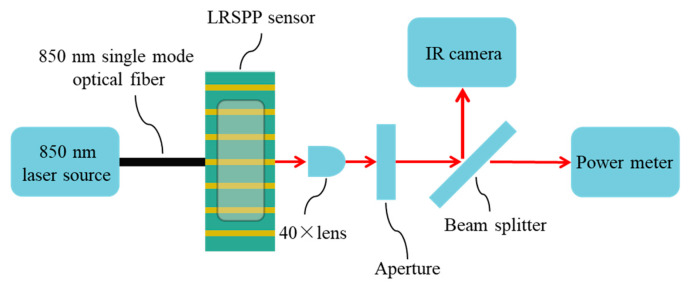
Schematic diagram of setup for characterizing LRSPP waveguide sensors.

To confirm the optical transmission performance of waveguides, the propagation loss of fully cladded LRSPP waveguides was measured at λ_0_ = 850 nm, using the setup sketched in Figure 12. A polarization-maintaining single-mode optical fiber was butt-coupled to the input of an Au stripe, launching 850 nm light from a laser and exciting the LRSPP mode. A 40× objective was used to collimate the optical output from the LRSPP waveguide. The background radiation was blocked by a variable aperture before the light beam was allowed to pass through a 50:50 beam splitter. An infrared (IR) camera was used to capture the mode field pattern. A power meter was used to measure the output power.

**Figure 13:**
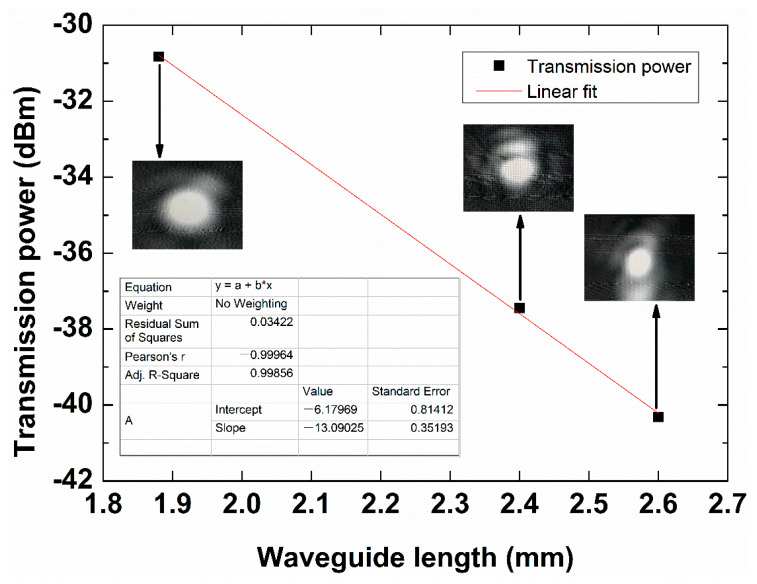
Transmission power as a function of LRSPP waveguide length at optical wavelength 850 nm. Linear fitting of the measured insertion loss of LRSPP waveguides with different lengths yields a mode power attenuation MPA*_w_* of 13.09 dB/mm at an adjusted R-squared of 0.99856 and a standard deviation of 0.814. Insets show the far field LRSPP mode patterns.

Following the cut-back method, the optical power emerging from 1.5 μm wide, 25 nm thick Au stripe waveguides, of lengths of 1.88, 2.40, and 2.60 mm, was measured. As shown in Figure 13, the insets show the captured far-field mode pattern. Linear fitting of the measured insertion loss of LRSPP waveguides with different lengths yields a mode power attenuation MPA*_w_* of 13.09 dB/mm at an adjusted R-squared of 0.99856, and a standard deviation of 0.814, which coincides well with the theoretical prediction of 12.01 dB/mm. The measured coupling loss of about 4.62 dB/facet is also close to the theoretical value of 3.92 dB/facet. During simulation, the fiber core diameter was chosen as 4.4 μm. The difference between the experimental results and theoretical expectations lies in the non-ideal waveguide end-facet at the input, which causes excess coupling loss. The facets were producing by slicing a wafer using a sharp knife (facets used as produced, unpolished). The coupling loss can be reduced by introducing dicing and polishing processes to obtain better facets. Nevertheless, this measurement supports the sensor design and the theoretical analysis, implying that the sensor design has good practical feasibility.

## 6. Conclusions

A bulk RI sensor based on a straight LRSPP waveguide is theoretically proposed. The polymer SU-8 was adopted as the cladding material to realize high-RI sensing. The LRSPP modal characteristics of Au stripes, and its geometric parameters, were investigated numerically via the FDE method at the optical free-space wavelength of 850 nm. The best bulk sensitivity can be obtained with a 1.5 μm wide, 25 nm thick, straight LRSPP waveguide. Fabricated waveguides were characterized, supporting the feasibility of the proposed design and validating the numerical modelling. This sensor has potential as a portable, low-cost refractometer.

## Figures and Tables

**Table 1:** Mode power distribution in SU-8, Au-stripe, and analyte.

*n_a_*	Power in Au (%)	Power in SU-8 (%)	Power in Analyte (%)	MPA (dB/mm)	Sensitivity (dB/RIU/mm)
1.562	0.048	58.795	41.181	14.36	196
1.566	0.044	55.089	44.862	13.00	
1.570	0.042	52.331	47.597	12.21	
1.574	0.041	49.992	49.992	12.01	
1.578	0.042	47.637	52.290	12.35	188
1.582	0.045	45.058	54.917	13.15	
1.586	0.048	41.737	58.221	14.25	

**Table 2:** RI sensing performance comparison.

Platform	Structure	RI Range	Sensitivity	Wavelength	Size	Ref
Fiber	Tapered core	1.3–1.4	8000%/RIU	632 nm	NA	[29]
Fiber	Tapered core	1.4304–1.4320	19,212.5 nm/RIU	1474–1615 nm	NA	[30]
POF	Tapered core	1.33–1.41	950 μW/RIU @633 nm	532, 633, and 780 nm	NA	[31]
Hybrid-plasmonic	Straight waveguide	1.3306–1.3326	2 × 10^5^ dB/RIU	632.8 nm	75 μm	[32]
Hybrid-plasmonic	Dual slot waveguide	10–100% IPA	1061 nm/RIU	1550 nm	40 μm	[33]
LRSPR	Prism	1.518–1.576	2000–6600 nm/RIU	400–800 nm	NA	[34]
This work	Straight waveguide	1.562–1.586	196 dB/RIU/mm	850 nm	<3 mm	

## References

[1] Fan H., Buckley R., Berini P. (2012). Passive long-range surface plasmon-polariton devices in Cytop. Appl. Opt..

[2] Berini P. (2008). Bulk and surface sensitivities of surface plasmon waveguides. New J. Phys..

[3] Breukelaar I., Charbonneau R., Berini P. (2006). Long-range surface plasmon-polariton mode cutoff and radiation in embedded strip waveguides. J. Appl. Phys..

[4] Wong W., Adikan F., Berini P. (2015). Long-range surface plasmon Y-junctions for referenced biosensing. Opt. Express.

[5] Fan H., Berini P. (2018). Bulk sensing using a long-range surface-plasmon triple-output Mach-Zehnder interferometer. IEEE J. Lightwave Technol..

[6] Khan A., Krupin O., Lisicka-Skrzek E., Berini P. (2013). Mach-Zehnder refractometric sensor using long-range surface plasmon waveguides. Appl. Phys. Lett..

[7] Wong W., Berini P., Fan H., Adikan F.R.M. (2018). Multichannel long-range surface plasmon waveguides for parallel biosensing. IEEE J. Lightwave Technol..

[8] Oleksiy K., Hamoudi A., Chen W., Tait R.N., Berini P. (2013). Biosensing using straight long-range surface plasmon waveguides. Opt. Express.

[9] Schmidt S., Flueckiger J., Wu W., Grist S.M., Fard S.T., Donzella V., Khumwan P., Thompson E.R., Wang Q., Kulik P. (2014). Improving the performance of silicon photonic rings, disks, and bragg gratings for use in label-free biosensing. SPIE.

[10] Salleh M., Glidle A., Sorel M., Reboud J., Cooper J. (2013). Polymer dual ring resonators for label-free optical biosensing using microfluidics. Chem. Commun..

[11] Halldorsson J., Arnfinnsdottir N., Jonsdottir A., Agnarsson B., Leosson K. (2010). High index contrast polymer waveguide platform for integrated biophotonics. Opt. Express.

[12] Wang X., Sun J., Liu Y., Sun J.-W., Chen C.-M., Sun X.-Q., Wang F., Zhang D.-M. (2014). 650-nm 1 × 2 polymeric thermo-optic switch with low power consumption. Opt. Express.

[13] Kou L., Labrie D., Chylek P. (1993). Refractive indices of water and ice in the 0.65-to 2.5-µm spectral range. Appl. Opt..

[14] Slavik R., Homola J. (2008). Ultrahigh resolution long range surface plasmon-based sensor. Sens. Actuators B Chem..

[15] Joo Y., Song S., Magnusson R. (2010). Demonstration of long-range surface plasmon-polariton waveguide sensors with asymmetric double electrode structures. Appl. Phys. Lett..

[16] Wark A., Lee H., Corn R. (2005). Long-range surface plasmon resonance imaging for bioaffinity sensors. Anal. Chem..

[17] Ji L., Sun X., He G., Liu Y., Wang X., Yi Y., Chen C., Wang F., Zhang D. (2017). Surface plasmon resonance refractive index sensor based on ultraviolet bleached polymer waveguide. Sens. Actuators B Chem..

[18] Ji L., Sun X., Yang G., Sun X., Yi Y., Wang X., Chen C., Zhang D. (2017). SU-8 grating assisted intermodal interference in surface plasmon polariton waveguide. Opt. Mater. Express.

[19] Huang S., Lai C., Sheu F., Tsai W.-S. (2017). Characterization of long-range plasmonic waveguides at visible to near-infrared regime. AIP Adv..

[20] Prajzler V., Nekvindova P., Hyps P., Lyutakov O., Jeřábek V. (2014). Flexible polymer planar optical waveguides. Radio Eng..

[21] Yakubovsky D., Arsenin A., Stebunov Y., Fedyanin D.Y., Volkov V.S. (2017). Optical constants and structural properties of thin gold films. Opt. Express.

[22] Berini P. (2007). Long-range surface plasmon-polariton waveguides in silica. J. Appl. Phys..

[23] Liu W., Yan J., Shi Y. (2017). High sensitivity visible light refractive index sensor based on high order mode Si_3_N_4_ photonic crystal nanobeam cavity. Opt. Express.

[24] Cai H., Yang Y., Chen X., AliMohammad M., Ye T.-X., Guo C.-R., Yi L.-T., Zhou C.-J., Liu J., Ren T.-L. (2015). A third-order mode high frequency biosensor with atomic resolution. Biosens. Bioelectron..

[25] Jhonattan C., Gabrielli L.H., Lechuga L.M., Figueroa H.E.H. (2019). Trimodal waveguide demonstration and its implementation as a high order mode interferometer for sensing application. Sensors.

[26] Charbonneau R., Scales C., Breukelaar I., Fafard S., Lahoud N., Mattiussi G., Berini P. (2006). Passive integrated optics elements based on long-range surface plasmon polaritons. IEEE J. Lightwave Technol..

[27] Wong W., Krupin O., Mahamd A., Berini P. (2015). Optimization of long-range surface plasmon waveguides for attenuation-based biosensing. IEEE J. Lightwave Technol..

[28] Wong W., Mahamd A., Berini P. (2014). Surface sensitivity of straight long-range surface plasmon waveguides for attenuation-based biosensing. Appl. Phys. A.

[29] Tai Y., Wei P. (2010). Sensitive liquid refractive index sensors using tapered optical fiber tips. Opt. Lett..

[30] Mallik A. (2015). High sensitivity refractive index sensor based on a tapered small core single-mode fiber structure. Opt. Lett..

[31] Feng D., Liu G., Liu X., Jiang M.-S., Sui Q.-M. (2014). Refractive index sensor based on plastic optical fiber with tapered structure. Appl. Opt..

[32] Madaan D., Kapoor A., Sharma V. (2018). Ultrahigh sensitivity plasmonic refractive-index sensor for aqueous environment. IEEE Photon. Technol. Lett..

[33] Sun X., Dai D., Thylén L., Wosinski L. (2015). High-sensitivity liquid refractive-index sensor based on a Mach-Zehnder interferometer with a double-slot hybrid plasmonic waveguide. Opt. Express.

[34] Jiang Y., Liu B., Zhu X., Tang X.-L., Shi Y.-W. (2015). Long-range surface plasmon resonance sensor based on dielectric/silver coated hollow fiber with enhanced figure of merit. Opt. Lett..

[35] Vernoux C., Chen Y., Markey L., Spârchez C., Arocas J., Felder T., Neitz M., Brusberg L., Weeber J., Bozhevolnyi S.I. (2018). Flexible long-range surface plasmon polariton single-mode waveguide for optical interconnects. Opt. Mater. Express.

[36] Tang J., Liu Y., Zhang L., Fu X.-C., Xue X.-M., Qian G., Zhao N., Zhang T. (2018). Flexible thermo-optic variable attenuator based on long-range surface plasmon-polariton waveguides. Micromachines.

